# Refinando o Risco Cardiovascular: Olhando Abaixo da Superfície do Cálcio

**DOI:** 10.36660/abc.20220763

**Published:** 2022-11-23

**Affiliations:** 1 The George Washington University School of Medicine Divisão de Cardiologia Washington DC EUA Divisão de Cardiologia, The George Washington University School of Medicine, Washington, DC – EUA; 2 Vrije Universiteit Amsterdam Amsterdam UMC Departamento de Cardiologia Amsterdam Holanda Departamento de Cardiologia, Amsterdam UMC, Vrije Universiteit Amsterdam, Amsterdam – Holanda; 3 University of Amsterdam Amsterdam UMC Departamento de Medicina Vascular Amsterdam Holanda Departamento de Medicina Vascular, Amsterdam UMC, University of Amsterdam, Amsterdam – Holanda

**Keywords:** Angiografia por Tomografia Computadorizada, Gordura Epicárdica, Cálcio da Artéria Coronária, Placa, Aterosclerose

A doença cardiovascular aterosclerótica (DCVA) é uma doença sistêmica iniciada por um influxo endotelial de partículas lipídicas, incluindo lipoproteínas de baixa densidade (LDL), com subsequente ativação endotelial via recrutamento local de células inflamatórias.^1^ Esse processo local– desencadeado pela exposição determinada pela idade a fatores de risco da DCVA genéticos, ambientais e de estilo de vida– é o primeiro passo de um processo que levará a um estado inflamatório sistêmico crônico e de baixo grau.^2^ A exposição prolongada do endotélio aos fatores de risco DCVA e este estado inflamatório aumentará o número de placas vulneráveis e pode eventualmente levar à ruptura da placa resultando em eventos DCVA.

Vários esforços têm se concentrado em medir a inflamação sistêmica e endotelial. Ridker et al. mostraram que os níveis de proteína C-reativa (PCR) foram positivamente associados a eventos futuros de DCVA.^3^ Ensaios subsequentes, incluindo a justificativa para o uso de estatinas na prevenção: um ensaio de intervenção avaliando a rosuvastatina (JUPITER), provaram a existência de um importante componente de inflamação residual segmentável (posteriormente denominado inflamassoma NLRP3) através do tratamento com rosuvastatina.^4^ Na imagem, os pesquisadores do Framingham Heart Study foram os primeiros a mostrar que os volumes de gordura pericárdica e visceral de tomografias computadorizadas sem contraste estavam associados a níveis aumentados de marcadores inflamatórios como PCR e IL-6 como fatores de risco independentes para DCVA.^5^ Usando angiografia por TC coronária (ATCC), descobriu-se que a medição da densidade do tecido adiposo pericoronário (DTAP) - semelhante à inflamação da gordura perivascular - forneceu valor discriminatório adicional para prever eventos de DCVA, independentemente das características da placa inflamatória de alto risco.^6^

Nesta edição de *Arquivos de Brasileiros de Cardiologia*, Martins et al. testaram a relação entre volume de gordura epicárdica, função endotelial e cálcio da artéria coronária (CAC) em 470 participantes do Estudo Longitudinal de Saúde do Adulto (ELSA-Brasil) que realizaram tomografia computadorizada sem contraste.^7^ O volume de gordura epicárdica foi avaliado usando um método totalmente automático e validado antes da calibração manual usando o MeVisLab,^8^ enquanto a função endotelial foi avaliada por tonometria de artéria periférica. Em uma coorte relativamente jovem com idade média de 55 anos, os autores descobriram que o volume de gordura epicárdica estava associado a múltiplos fatores de risco, incluindo idade avançada, sexo masculino, circunferência da cintura e triglicerídeos. O principal achado deste estudo foi que o volume de gordura epicárdica não foi associado com CAC, mas com disfunção endotelial em análises multivariáveis.

Esses achados, exclusivos da coorte brasileira estudada, contribuem de forma incremental para o entendimento de que os depósitos de tecido adiposo extravascular (pericárdico) e a inflamação estão associados à disfunção endotelial intravascular e à inflamação. Em particular, a ausência de uma relação profunda com CAC, que também foi observada em vários outros estudos,^9^ sugere vias distintas de risco aterosclerótico e fundamenta a necessidade de olhar além do escore de cálcio da artéria coronária do paciente, que representa um marcador de placa estável. A pontuação CAC fornece uma estimativa aproximada da quantidade total de placa presente; ao contrário da ATCC, ela não pode detectar a carga de placas não calcificadas nem distinguir a placa vulnerável das lesões calcificadas de maior densidade e menor risco, que podem representar a estabilidade da placa do tratamento.^10,11^

Identificar disfunção endotelial e doença microvascular por meio de imagens sem contraste é uma estratégia atraente, principalmente em regiões onde os métodos não invasivos mais recentes não estão disponíveis. Tais métodos para medir a disfunção endotelial intravascular incluem tomografia por emissão de pósitrons de estresse (TEP) para medir quantitativamente o fluxo sanguíneo miocárdico e ressonância magnética cardiovascular (RMC) com gadolínio para medir o índice de reserva de perfusão miocárdica.^12^ Na ausência dessas técnicas, bem como da DTAP derivada da ATCC, a medição do volume de gordura epicárdica a partir de imagens de TC sem contraste pode fornecer informações adicionais de estratificação de risco importantes sobre inflamação endotelial, disfunção e presença de placa vulnerável, além de analisar apenas CAC.^13^

Os autores estão de parabéns pelo estudo realizado. É importante reconhecer, no entanto, as evidentes limitações do trabalho. Primeiro, dada a população de estudo relativamente jovem, 56% dos pacientes neste estudo não apresentavam cálcio nas artérias coronárias, questionando o poder da análise para excluir uma relação entre o volume de gordura epicárdica e o cálcio nas artérias coronárias. Em contraste com a análise multivariada ajustada para vários fatores de risco DCVA, a análise univariada do presente estudo mostrou que pacientes com volume de gordura epicárdico acima da mediana apresentaram mais CAC. Embora as evidências disponíveis sejam conflitantes, parece haver pelo menos uma associação modesta entre o volume de gordura epicárdica e o escore de cálcio da artéria coronária em estudos maiores.^14^ Ainda assim, quando adicionado a um escore de risco composto por CAC, o volume de gordura epicárdica melhorou significativamente a predição de doença arterial coronariana obstrutiva; em um estudo de Zhou et al. em 5.743 pacientes, confirmando o potencial do volume de gordura epicárdica além da pontuação CAC.^13^ Em segundo lugar, o método de tonometria arterial periférica usado neste estudo é uma medida substituta da (dis)função endotelial – em oposição ao padrão-ouro de vasorreatividade coronariana após acetilcolina intracoronária – e também é afetado por fatores externos, como a ativação do sistema nervoso autônomo.^15^ Portanto, permanece desconhecido se as alterações observadas na razão da tonometria da artéria periférica refletem disfunção endotelial ou apenas aumento do tônus simpático em pacientes de alto risco. Por fim, o estudo não correlaciona o volume de gordura epicárdica aos desfechos clínicos. Coletivamente, dadas as limitações e a pequena coorte, o estudo deve ser interpretado como promissor, intrigante, mas gerador de hipóteses.

Em resumo, a aterosclerose é uma doença sistêmica, multifatorial e complexa, cujas características não podem ser capturadas em uma única métrica. Seja usando imagens com contraste ou sem contraste, deve-se olhar abaixo da superfície do cálcio - identificando inflamação e disfunção endotelial resultando em componentes de placa de alto risco não calcificados - para aumentar a precisão na estratificação de risco de DCVA.
