# Supplementary material for: Refinando o Risco Cardiovascular: Olhando Abaixo da Superfície do Cálcio
Source: Arq Bras Cardiol. 2022 Nov 23;119(6):921–2. [Article in Portuguese] doi: 10.36660/abc.20220763 (PMC9814812; doi:10.36660/abc.20220763)
Supplement: Translation

# Translation: Refining Cardiovascular Risk: Looking Beneath the Calcium Surface

**Affiliations:** 1 Washington DC USA Divisão de Cardiologia, The George Washington University School of Medicine, Washington, DC – USA; 2 Amsterdam Netherlands Departamento de Cardiologia, Amsterdam UMC, Vrije Universiteit Amsterdam, Amsterdam – Netherlands; 3 Amsterdam Netherlands Departamento de Medicina Vascular, Amsterdam UMC, University of Amsterdam, Amsterdam – Netherlands

**Keywords:** Coronary Computed Tomography Angiography, Epicardial Fat, Coronary Artery Calcium, Plaque, Atherosclerosis

Atherosclerotic cardiovascular disease (ASCVD) is a systemic disease initiated by an endothelial influx of lipid particles, including low-density lipoproteins (LDL), with subsequent endothelial activation via local recruitment of inflammatory cells.^1^ This local process – elicited by age-determined exposure to genetic, environmental, and lifestyle ASCVD risk factors – is the first step of a process that will lead to a chronic, low-grade systemic inflammatory state.^2^ Prolonged exposure of the endothelium to ASCVD risk factors and this inflammatory state will increase the number of vulnerable plaques and may eventually lead to plaque rupture resulting in ASCVD events.

Multiple efforts have focused on measuring systemic and endothelial inflammation. Ridker et al. showed that C-reactive protein (CRP) levels were positively associated with future ASCVD events.^3^ Subsequent trials, including the Justification for the Use of Statins in Prevention: an Intervention Trial Evaluating Rosuvastatin (JUPITER), proved the existence of a targetable, important residual inflammation component (later denoted as the NLRP3 inflammasome) through treatment with rosuvastatin.^4^ On the imaging side, investigators from the Framingham Heart Study, were the first to show that pericardial and visceral fat volumes from non-contrast CT scans were associated with increased levels of inflammatory markers such as CRP and IL-6 as independent risk factors for ASCVD.^5^ Using coronary CT angiography (CCTA), it was discovered that measurement of pericoronary adipose tissue (PCAT) density – resembling perivascular fat inflammation – provided additional discriminatory value for predicting ASCVD events, independent from inflammatory high-risk plaque features.^6^

In this issue of *Arquivos de Brasileiros de Cardiologia*, Martins and colleagues tested the relation between epicardial fat volume, endothelial function and coronary artery calcium (CAC) among 470 participants from the Brazilian Longitudinal Study of Adult Health (ELSA-Brasil) who underwent non-contrast CT imaging.^7^ Epicardial fat volume was assessed using a fully automatic, validated method before manual calibration using MeVisLab,^8^ while the endothelial function was assessed using peripheral artery tonometry. In a relatively young cohort with a mean age of 55, the authors found that epicardial fat volume was associated with multiple risk factors, including older age, male sex, waist circumference and triglycerides. The main finding of this study was that epicardial fat volume was not associated with CAC but with endothelial dysfunction in multivariable analyses.

These findings, unique to the studied Brazilian cohort, add incrementally to an understanding that extravascular (pericardial) adipose tissue deposits and inflammation are associated with intravascular endothelial dysfunction and inflammation. In particular, the absence of a profound relationship with CAC, which was also observed in several other studies,^9^ suggests distinct pathways of atherosclerotic risk and underlies the need to look beyond the patient’s coronary artery calcium score alone, which represents a marker of stable plaque. CAC scoring provides a rough estimate of the amount of total plaque present; unlike CCTA, it cannot detect non-calcified plaque burden nor distinguish vulnerable plaque from the highest density, lower risk calcified lesions, which may represent plaque stability from treatment.^10,11^

Identifying endothelial dysfunction and microvascular disease through non-contrast imaging is an attractive strategy, particularly in regions where newer non-invasive methods are unavailable. Such methods to measure intravascular endothelial dysfunction include stress positron emission tomography (PET) to quantitatively measure myocardial blood flow and gadolinium-enhanced cardiovascular magnetic resonance (CMR) imaging to measure the myocardial perfusion reserve index.^12^ in the absence of these techniques as well as the CCTA-derived PCAT, measurement of epicardial fat volume from non-contrast CT imaging could provide important additive risk-stratifying information on endothelial inflammation, dysfunction, and presence of vulnerable plaque beyond solely analyzing CAC.^13^

The authors are to be congratulated on the performed study. It is important to recognize, however, the evident limitations of the work. First, given the relatively young study population, 56% of patients in this study had no coronary artery calcium present, questioning the power of the analysis to exclude a relationship between epicardial fat volume and coronary artery calcium. In contrast to the multivariate analysis adjusted for several ASCVD risk factors, the univariate analysis of the current study showed that patients with above-median epicardial fat volume did have more CAC. Although the available evidence is conflicting, there seems to be at least a modest association between epicardial fat volume and coronary artery calcium score in larger studies.^14^ Still, when added to a risk score comprising CAC, epicardial fat volume significantly improved the prediction of obstructive coronary artery disease; in a study by Zhou et al. in 5743 patients, confirming the potential of epicardial fat volume beyond CAC scoring.^13^ Second, the peripheral arterial tonometry method used in this study is a surrogate measure of endothelial (dys)function – as opposed to the gold standard of coronary vasoreactivity after intracoronary acetylcholine – and is also affected by external factors such as autonomic nervous system activation.^15^ Therefore, it remains unknown whether the observed changes in peripheral artery tonometry ratio reflect endothelial dysfunction or merely increased sympathetic tonus in high-risk patients. Finally, the study does not correlate epicardial fat volume to clinical outcomes. Collectively, given the limitations and small cohort, the study should be interpreted as promising, intriguing, but hypothesis-generating.

In summary, atherosclerosis is a systemic, multifactorial, complex disease whose characteristics cannot be captured in a single metric. Whether using contrast or non-contrast imaging, one must look beneath the calcium surface - identifying endothelial inflammation and dysfunction resulting in non-calcified high-risk plaque components - to enhance precision in ASCVD risk stratification.
